# Supplementary material for: Host and viral determinants of airborne transmission of SARS-CoV-2 in the Syrian hamster
Source: eLife. 2024 Feb 28;12:RP87094. doi: 10.7554/eLife.87094 (PMC10942639; doi:10.7554/eLife.87094)
Supplement: Supplementary file 1. — Donor animals (N=7) were inoculated with either the Alpha or Delta variant and paired together randomly in 7 attack rate scenarios (A-G). One day after inoculation, 4–5 sentinels were exposed for a duration of 4 h in an aerosol transmission set-up. Percentage of Alpha and Delta detected in oropharyngeal swabs taken at day 2 and day 5 post exposure by deep sequencing. [file elife-87094-supp1.docx]

| **Percentage reads (%) mapped to Delta** | | | |
| --- | --- | --- | --- |
| Cage | Sentinel | Oral swab day | |
|  | | 3 | 5 |
| A | A1 | 0 | 0 |
|  | A2 | 0 | 0 |
|  | A3 | 0 | 0 |
|  | A4 | 0 | 0 |
|  | A5 | 0 | 0 |
| B | B1 | 0 | 0 |
|  | B2 | 0 | 0 |
|  | B3 |  |  |
|  | B4 | 0 | 0 |
|  | B5 | 97 | 96.667 |
| C | C1 |  | 96.333 |
|  | C2 |  | 0 |
|  | C3 |  | 96.333 |
|  | C4 | 0 | 0 |
|  | C5 | 97.333 | 96.667 |
| D | D1 |  |  |
|  | D2 |  | 97 |
|  | D3 | 96.667 | 97 |
|  | D4 |  | 97 |
|  | D5 |  |  |
| E | E1 | 97 |  |
|  | E2 |  |  |
|  | E3 | 97.333 | 97.333 |
|  | E4 |  |  |
|  | E5 |  | 0 |
| F | F1 | 0 | 0 |
|  | F2 | 0 | 0 |
|  | F3 | 97 | 90.333 |
|  | F4 | 0 | 10.5 |
|  | F5 | 96.667 | 93.667 |
| G | G1 |  | 0 |
|  | G2 |  | 97.333 |
|  | G3 |  |  |
|  | G4 |  |  |
